# A Perspective on Using Organic Molecules Composing Carbon Dots for Cancer Treatment

**DOI:** 10.7150/ntno.80076

**Published:** 2023-01-22

**Authors:** Musbahu Adam Ahmad, Yu-yu Aung, Alfa Akustia Widati, Satya Candra Wibawa Sakti, Sri Sumarsih, Irzaman Irzaman, Brian Yuliarto, Jia-yaw Chang, Mochamad Zakki Fahmi

**Affiliations:** 1 Department of Chemistry Universitas Airlangga, Surabaya 60115, Indonesia.; 2 Supramodification Nano-micro Engineering (SPANENG) research group, Universitas Airlangga, Surabaya 60115, Indonesia.; 3 Department of Physics, Bogor Agricultural University of Indonesia, Bogor16680, Indonesia.; 4 Advanced Functional Materials Laboratory, Department of Engineering Physics, Institut Teknologi Bandung, Bandung40132, Indonesia.; 5 Department of Chemical Engineering, National Taiwan University of Science and Technology, Taipei, 10607, Taiwan, ROC.

**Keywords:** Carbon dots, cancer therapy, organic compounds, natural product, nanomedicine

## Abstract

Fluorescent Carbon dots (CDs) derived from biologically active sources have shown enhanced activities compared to their precursors. With their prominent potentiality, these small-sized (<10nm) nanomaterials could be easily synthesized from organic sources either by bottom-up or green approach. Their sources could influence the functional groups present on the CDs surfaces. A crude source of organic molecules has been used to develop fluorescent CDs. In addition, pure organic molecules were also valuable in developing practical CDs. Physiologically responsive interaction of CDs with various cellular receptors is possible due to the robust functionalization on their surface. In this review, we studied various literatures from the past ten years that reported the potential application of carbon dots as alternatives in cancer chemotherapy. The selective cytotoxic nature of some of the CDs towards cancer cell lines suggests the role of surface functional groups towards selective interaction, which results in over-expressed proteins characteristic of cancer cell lines. It could be inferred that cheaply sourced CDs could selectively bind to overexpressed proteins in cancer cells with the ultimate effect of cell death induced by apoptosis. In most cases, CDs-induced apoptosis directly or indirectly follows the mitochondrial pathway. Therefore, these nanosized CDs could serve as alternatives to the current kinds of cancer treatments that are expensive and have numerous side effects.

## Introduction

Carbon-based nanomaterials having lower than 10 nm diameter, known as carbon dots, have caught the attention of researchers since their unprecedented discovery in 2004 1. Since then, Carbon dots (CDs) have been named with different terms. They were called carbon quantum dots (CQDs), polymer dots (PD), carbon nanodots (CND), graphene quantum dots (GQDs) 2,3, or even graphene-oxide quantum dots (GOQDs) 4. Semeniuk et al. 3 discussed a detailed description of these terms. Bayda*et al.* 2 also mentioned such terms briefly. In this review, we would use the term “carbon dots” abbreviated “CDs” to address all the terms mentioned earlier generally. To date, their potential use in cancer therapy has been brought to light. The realization of their photoluminescence properties upon surface passivation by Sun *et al.* 5 unveiled their potential applications involving different fields of interest. The potential advantage of these fluorescent carbon dots for bioimaging *in vitro* and *in vivo* has been demonstrated 6-10. Carbon dots (CDs), compared to their silicon counterparts, possess an encouraging photoluminescence nature coupled with the added advantages of significantly lower cytotoxicity and excellent biocompatibility, making its usage for cellular staining possible. Potency in cancer diagnosis using carbon dots was reported. In the research, MCF-7 breast cancer cells were imaged, labeling the cell membrane and cytoplasm 7. These CDs enter the cell in a temperature-dependent manner; at about 4ºC, limited entrance was observed 7. Recently, Fahmi *et al.* 10 have shown that CDs could also enter the cell via endocytosis, as earlier hypothesized 7. As such, CDs capable of interacting with the cell's membrane surface could enter even at 4ºC. The influence that surface functional groups on CDs have on cellular penetration and interaction and its tunability into cellular recognitive 11 materials have paved the way for more detailed studies on the future biomedical applications of carbon dots 12. In addition to its use for imaging gastric cancer cell lines (MGC-803), CDs modified with ribonuclease A (RNase A@CDs) showed potency as anti-cancer candidates due to the concentration-dependent cytotoxic effect shown by RNase A@CDs. Thus, suggesting its use in cancer therapy 13. CDs from ginger juice could selectively suppress the growth of human hepatocellular carcinoma cells (hepG2) 14. These and many other CDs from different sources are useful in cancer therapy 11,15,16. Although not thoroughly studied, CDs with potential procoagulant activity have been reported 17,18. In addition, HIV-1 infection was hindered by the employment of CDs based on aminophenyl boronic acids 19, thus can be potential for HIV treatment. Besides their application for bio-imaging and therapy, CDs have other applications as biosensors. It was demonstrated that CDs from hydroxypropylmethyl cellulose could be used as sensors for ciprofloxacin detection 20. A highly sensitive and selective molecularly imprinted polymer-based CDs (MIP@CDs) was prepared for the detection of acetyl cholinesterase (AChE), an essential enzyme in the diagnosis of Alzheimer's disease (AD) 21. In addition, Fe_3_O_4_@Carbon dot nanocomposites were used as curcumin carriers for possible AD treatment 22.

On the other hand, CDs have been utilized as initiators for low-cost, non-toxic, and one-step polymerization of pyrrole and its copolymer 23. Furthermore, the sensing ability of CDs towards metal ions was reported 24,25. Campuzano *et al.* 26 cited broader applications of CDs in biosensing. They were used to detect glucose, H_2_O_2,_ dopamine, and TNT. These CDs were also involved in preparing DNA sensors to detect single gene mutation 26. CDs are primarily used as organic molecule detectors by further immobilizing enzymes at their surface. For instance, horseradish peroxidase immobilized at the surface of CDs was used to detect hydrogen peroxide, while glucose oxidase immobilized at the surface of CDs could be used to detect glucose. Similar to the above discussed, these CDs' surface functionalization plays a vital role in enzyme immobilization 27,28. In summary, the wide range of potential applications shown by carbon dots could be traced back to their amenable nature for surface modification. Therefore, it is crucial to consider the engineering of CDs surfaces for broader and more practical applications.

Several reviews emphasized carbon dots' application for cancer therapy 29-32. However, all lack detailed studies on carbon dots' natural cytotoxicity towards cancer cells with few impacts on normal cells. Thus, more emphasis is placed herein on studying carbon dots that show the independent ability to confer cytotoxicity to cancer cells. This condition confirmed that types of carbon dots (1) could be easily synthesized from a range of available sources, (2) do not necessarily require the influence of other factors like laser light as in photothermal therapy for their application, (3) could therefore be used directly for cancer therapy as an alternative to chemotherapy. Therefore, this review will analyze and obtain literature from 2012 to 2023 from PubChem, Scopus, and Web of Science databases. Also, the keywords for the search are “carbon dots cancer”, “carbon dots anti-cancer”, and “carbon dots anti-tumor” or “carbon dots anti-cancer natural product”. All data were chosen based on the said criteria above and on the fact that the CDs are synthesized from biologically critical organic molecules/sources plus being able to showcase innate specific toxicity towards tumors. The review comprises sections covering the developments in carbon dots synthetic methods and an overview of CDs synthesized from organic sources. Furthermore, the anti-cancer potency of these CDs will be explored, and then the review would proceed with discussions about the mechanism of interaction between carbon dots and cancer cells.

## Advances in Carbon Dots Synthesis

Owing to their immense potency for various applications, CDs are continuously prepared and tested for different applications. An essential aspect of their ability to show vast potency is the methods employed for their synthesis. Therefore, discussing the methods of carbon dots synthesis is as important as discussing their applications. Under this circumstance, several studies reported synthetic methods of carbon dots 32-37. Therefore, synthesis methods of carbon dots would not be discussed immensely. Nevertheless, however, we would briefly discuss the general overview of the carbon dots synthesis with the green synthetic approach put into consideration as well. Nanomaterials, in general, are synthesized by two broad approaches, i.e., the Top-Down approach and the Bottom-Up approach. Carbon dots are no exceptions. These two approaches are used to synthesize them. Furthermore, when sustainable and less toxic sources are used for either of the two approaches, the synthesis method is considered “Green” 38. Therefore, more discussion regarding these synthetic approaches is made in this section.

## Top-Down Approach

The first reported synthesis of Carbon dots was achieved by electrophoretic remission of single-walled carbon nanotubes (SWCNTs) 1. Many synthetic methods used are classified onto two approaches. The top-down approach is based on converting a bulky precursor into small-sized (<10 nm) CDs. Several techniques could achieve this approach, including fragmentation using a laser 39, selective oxidation of the bulk material using hydrogen peroxide 40,41, as well as oxidation using a mixture of strong acids (H_2_SO_4_ and HNO_3_) 42,43.

While laser ablation involves directing the laser light on a starting material which eventually leads to fragmentation of the bulk material 5,39, oxidation using hydrogen peroxide involves the pretreatment of carbon source into an ultrafine powder dispersed in H2O, followed by controlled addition of H2O2 and subsequent heating at 80ºC 40, or 120ºC 41 for 40 minutes. In addition, 41 showed the dependence of surface functionalization, sizes, and the quantum yield on the reaction time. This condition implies that the longer the reaction time, the smaller the CDs size obtained, thus, the better fluorescent cellular imaging 41. With regard to oxidation with a mixture of acids, the process involves mixing a few milligrams of the nanomaterial precursor and treating it with a 3:1 mixture of sulfuric and nitric acids followed by continuous heating and stirring for 15 hours 43 or 48 hours 42 at 110ºC or 140ºC respectively. The last report has shown that the optimal reaction condition occurs at 15 hours and 110ºC 43. Reactions beyond this optimum range yield no further increment in the yield 43. Thus, this affirms that optimization of reaction conditions is necessary to obtain approximately better properties. However, these methods are limited to small-scale production of CDs due to their low quantum yields. Also, the methods are expensive and require complex facilities 34.

## Bottom-Up Approach

The preparation of carbon dots by using small-sized precursors (molecules) has been reported. Generally, the method involves the use of molecules as carbon sources. The molecules undergo polymerization, dehydration, and carbonization 34 into the desired nanometer size range 19. Some of the techniques used to synthesize carbon dots via a bottom-up approach include hydrothermal 9,27,28,44,45, microwave 46,47, and pyrolysis 6,10,19 have been employed in the synthesis of Carbon dots.

Carbon dots from 2-amino-2-hydroxymethyl-propane-1,2-diol (TRIS), EDTA, Glycine and Cadaverine 45, polyacrylamide 28, chitosan 27, fruit juice and citric acid 9 were synthesized following hydrothermal method. In another approach, citric acid and 2-aminophenylboronic acid serve as carbon and nitrogen sources, respectively 19, catechin 6, tartaric acid/tyrosine 10, and the combined mixture of citric acid, boric acid, nitric acid, sulphuric acid and phosphoric acid 47 were used to synthesize CDs by pyrolysis method. Additionally, microwave-assisted synthesis was reported using citric acid combined with P, S, N, and B precursors to prepare N, P, B, S- doped CDs 47 and citric acid and urea 48. According to Sagbas and Sahiner 34, the microwave method is more favorable commercially faster and could happen within 5 minutes among all the methods under this category 34.

## Green Synthesis Approach

Synthesis of CDs is regarded as green when a less toxic starting material is used. Generally, the solvent and the precursor should exert minimal threat to the environment and the experimenter 38. A more detailed study on the green synthesis of carbon dots and their applications was previously reported 38. Considerable research involving the synthesis of CDs by the green approach has been reported in some literatures 40,44,49. The green synthesis may involve a top-down or bottom-up approach. The difference is seen in the starting materials and solvents used for the synthesis. If renewable and non-toxic, then the synthesis is considered to be green. However, if toxic or from a non-renewable source, the synthesis is not regarded as green. Green synthesis of fluorescent CDs by the hydrothermal method was reported using Orange Juice 49; *T. bispinosa* peels aqueous extract 50; milk 51; peanut shell 52; Garlic 53; *O. sanctum* 54; Rose-heart radish 55; walnut oil 56; spices 57; kiwi, avocado and pear fruits juice 9; Mulberry 58; Sweet lemon peel 44; and *Buchnania lannzan* 59 as green precursors. Though the hydrothermal method is widely used, other methods in the green synthesis of CDs were also reported. Microwave synthesis of CDs was done using aqueous glucose 60. The pyrolysis method was employed to synthesize peanut-derived 52 and Hair-derived CDs 61. Due to the enormous potential of these CDs, large-scale production is needed. Easy and less expensive means of preparation using renewable raw materials is a promising pathway in their large-scale production.

Generally, all three methods resulted in the successful production of CDs. However, higher emphasis is usually given to that method with more straightforward steps, lower energy and time consumption, and fewer safety concerns. Critically, it is evident that the top-down approach requires expensive and hazardous raw materials to produce CDs. Besides, the yields are too low and difficult to translate into large-scale production due to their production cost. Furthermore, it is not easy to control the size distribution of the CDs.

In contrast, the bottom-up approach offers a much easier means of preparing the CDs. For instance, microwave-assisted synthesis provides the required energy for the synthesis to occur within a short time. However, the problem with this approach is that it might lead to environmentally unfriendly by-products that might draw serious concerns. So, straight to the point, we could say that the synthesis of CDs by the green approach employing the bottom-up method could be more favorable in terms of cost of production, eco-friendliness, and simple means of preparation.

## Design of Carbon Dots from Low Molecular Weight (LMW) Organic Molecules

Besides the CDs synthetic method, choosing a precursor as the primary carbon source is another crucial aspect in this field. Similar to quantum dots, synthesizing carbon nanomaterials like carbon dots requires a material that could provide a carbon-rich core as it forms the nanomaterial. Carbon sources used in the synthesis includes inorganic sources like graphite rods 43,62, soot 63, graphene 39,43, carbon powder 42,43,64-66, coal 40,67, MWCNTs 68, Fullerene 41 and carbon nanotubes 1,43. Synthesis using these sources requires further surface passivation to obtain luminescent CDs and desired functional groups at the surface. Moreover, organic compounds/natural products have also been used to prepare these CDs. The compounds used could be pure or a mixture of compounds in a crude extract. For instance, citric acid is a widely used compound for preparing carbon dots. It could be singly used or in combination with one or more compounds. Furthermore, an organic source like the dried leaf of a plant could be used directly for its synthesis. The leaf may constitute different secondary metabolites contributing to the CDs' final structure. Synthesis of CDs using organic sources requires no further surface passivation to obtain luminescent CDs. Although 33 have reviewed the use of small organic molecules for CDs synthesis, we found it necessary to discuss similar coverage further. However, we further added natural products used in carbon dots synthesis into our discussion due to the amazing property shown by these CDs.

As mentioned above, a crude source may constitute many secondary metabolites and proteins. CDs have been prepared from various plant extracts or directly using plant parts. Interestingly, fruit juice was used to prepare CDs. The prepared CDs have about 1.5-4.5 nm size 49. *T. bispinosa* extract was used to prepare luminescent and biocompatible CDs with a size distribution between 5-10 nm for biological applications 50. Also, fluorescent CDs sourced from human hair with a mean diameter of 2.3 nm and size distribution from 3.2 to 8.8 nm have been reported to be potentially helpful for developing multidimensional fluorescent materials 61,69. Peanut shell-derived CDs were prepared and shown to have a mean diameter of 1.62 nm 52. Fresh *Ocimum sanctum* leaves were employed to synthesize CDs having sizes between 4-7 nm 54. Rose-heart radish CDs having an average diameter of 3.6 nm were applied for Fe^3+^ sensing and fluorescent imaging 55. Walnut oil produced CDs with an average diameter of 12.3±2.7 nm. 56. CDs derived from cinnamon, red chili, black pepper, and turmeric with their respective average diameters of 3.4±0.5, 3.1±0.2, 3.5±0.1, and 4.3±0.5 nm were synthesized 57. Juices from Kiwi, Avocado, and Pear were used to synthesize CDs, each with a size of around 4 nm 9. Furthermore, Mulberry leaves were used to prepare CDs with a size range of 2-4 nm 58. It could be deduced from previous literature that many organic sources, especially plant-based sources, could be used to produce CDs with varying structures and properties.

CDs prepared from distinct organic compounds have also been reported. Citric acid has been used as the primary precursor in synthesizing CDs. If further heteroatom doping and surface functionalization is desired on the CDs, the precursor of the heteroatom is added to the reaction mixture. For instance, citric acid-derived CDs with Nitrogen doping have been synthesized by adding urea 48,70, and ethylenediamine 71-73. CDs doped with multiple heteroatoms were reported as well. Doping with Nitrogen, Sulfur and Boron was reported using a mixture of citric acid and the dopants 3-aminophenylboronic acid (APBA) and thiourea as their respective precursors 74. Nevertheless, citric acid and the dopant APBA as both N and B sources were also crucial in the formation NBCDs 19. Single heteroatom doping was also reported using nitric acid for N doping, Boric acid for B doping, Sulphuric acid for S doping, and Phosphoric acid for P doping for unique synthesis 47. Likewise, combined gallic acid and citric acid were used to prepare CDs 75. Besides citric acid, CDs employing organic molecules as carbon precursors have also been reported. Photoluminescent CDs from Glycine molecules, ethylenediaminetetraacetic acid (EDTA), 2-amino-2-hydroxymethylpropane-1,3-diol (TRIS), and Cadaverine have also been reporte d^45^.Alanine and ethylenediamine were also reported in the preparation of CDs 76.

Furthermore, glycerol and silane prepared mitochondria imaging CDs 11. Antibacterial spermidine was used to produce CDs 77. Curcumin was also employed in CDs synthesis 78,79. Tryptophan based CDs were prepared for the delivery of drugs to the brain 80. Cancer-targeting biocompatible, photoluminescent CDs were prepared from folic acid 81. 1,4,5,8-tetraaminoanthraquinone and citric acid were used to prepare functionalized CDs 82. Another polyphenol, catechin, isolated from *Uncaria gambir,* was used to prepare CDs for tumor imaging 6. CDs synthesized from Glutathione were, nevertheless, reported 83,84. N-Hydroxypthalimide molecule was also used as the primary precursor for CDs 85. Tartaric acid and L-Tyrosine were used to prepare CDs 10. Monoethylene glycol (MEG) was used to prepare an enhanced CO2 hydrate inhibiting CDs 86. *Ortho* and *Para*-phenylenediamine were also used as precursors in CDs synthesis 87,88. Breviloquently, the synthesis of CDs from known organic sources was proven possible. Importantly, it should be noted that these sources could support dehydration and carbonization, as described by Kwee *et al.* 6. Thus, when choosing a compound with a known structure, it is important to consider its likelihood of undergoing dehydration for subsequent carbonization to occur, which is crucial for the formation of the CDs.

## Design of Carbon dots from Polymer Molecules

Besides being used as surface passivation on CDs 5,7,8, polymeric molecules are also not left behind in synthesizing CDs. These large molecules have been reported as precursors in CD development. Even though these are large molecules, their sizes do not translate to the size of the synthesized CDs. Carbon dots from polymeric molecules have been reported having varying applications as sensors 24,27,28,89-91, delivery systems 92-96, polymerization initiators 23, anti-cancer 97-99, cell imaging 100, as well as their use as anti-UV additives 101.

Polymeric precursors used for CDs synthesis includes those that were singly used as self-passivation and those used in combination with either small molecule like citric acid 100 or another polymer. Previously, PEG was employed as a surface passivator for CDs. This condition results in enhanced photoluminescence 5. More advanced synthesis was done using the PEG as the main carbon source, at the same time, self-passivator 23,102, and in combination with N and P sources 98. Moreover, polyethyleneimine (PEI) was employed for the preparation of CDs. These CDs have shown their usefulness as gene delivery systems 93,95,99 and for imaging human adenoid cystic carcinoma cells 100. Furthermore, 24,27,96 found the use of Chitosan in the synthesis of CDs for glucose sensing 27, metal ion detection 24, simultaneous imaging, and drug delivery 96. Carboxymethylcellulose (CMC) from palm oil sources carbon in prepared fluorescent CDs with a Nitrogen dopant, Linear-structured polyethyleneimine (LPEI). The prepared CDs were used for sensing Cu^2+^ ions 89. Lignin-derived CDs functionalized with coumarin derivatives were reported to have real-time cell visualization ability 103. Polyacrylamide-derived CDs were reported and involved in the electrochemical sensing of glucose 28. Porphyra polysaccharides were used as a carbon source for developing gene delivery CDs 92. CDs' tumor photothermal therapy (PTT) potential originated from polydopamine and folic acid precursors 97. Moreover, L-lysine polymer was used to synthesize CDs as detectors 90. Sodium alginate cross-linked by glutaraldehyde was used to develop CDs 101. Finally, CDs made from cellulose hydrogel were also reported 91.

In summary, large molecular weight organic molecules have also shown utility in developing CDs for various applications ranging from gene delivery to tumor therapy. Although it might be presumed that the sizes of CDs sourced from small or large molecules would depend on the starting material's size, literatures has shown the contrary 90,101. Furthermore, the size of CDs depends on the reaction conditions like temperature 101 or concentration of chemical carbonizing agent 90 between the precursor molecules. Therefore, monitoring the reaction conditions is extremely important in fluorescent CDs synthesis.

## Advantages of Organic Compounds/Natural Products based Carbon Dots for Cancer Treatment

As conventional chemotherapy remains ambiguous regarding its swift cancer treatment with no side effects, continuous research is being made to find better alternatives. It is, nevertheless, an expensive process and remains coupled with the possibility of re-development of another tumor post-treatment. Therefore, in this section, our discussion points to the direction where carbon dots could be used as alternatives to drugs used in chemotherapy. This section revolves around the possible use of only CDs for cancer treatment.

Carbon dots were synthesized for several purposes. Although these carbon dots could be thought to possess similar behavior, they were proven otherwise. Similar to organic compounds that show various applications regarding their structural features, carbon dots could be considered nanomaterials whose structure also influences their function. However, the difference is seen in their enhanced function compared to organic compounds. For instance, monoethylene glycol (MEG) inhibits CO_2_ hydration. Such a process is required for worthwhile CO_2_ sequestration. CDs synthesized from MEG showed better inhibition towards CO_2_ hydrate formation 86. Furthermore, resveratrol is a polyphenol that was shown to be capable of specific cancer targeting 104. When the resveratrol was used to form carbon dots, the resveratrol-sourced carbon dots showed better cytotoxicity toward cancer cells than the resveratrol itself 105. N-hydroxyphthalimide was shown to possess anti-tumor activity for the first time in 2016 106. The compound showed promising anti-cancer activity against breast cancer cell lines. Two years ago, the same N-hydroxyphthalimides were used to synthesize carbon dots with impressive anti-cancer activity against breast cancer cell lines. They were, furthermore, shown to be capable of attenuating the invasiveness and metastasis of these cell lines 85. Therefore, CDs behave similarly to organic compounds by showing different practical properties that relate to their structural arrangement. In addition, they seem to form a structure that enhances the property of their precursor molecules. Thus, they could be used for independent cancer therapy without needing external influencers, as in photothermal therapy or photodynamic therapy, thereby providing cheaper treatment. In the following section, we will classify CDs with anti-cancer potency based on the sources of their preparation. The sources would be classified as the crude source and pure organic sources.

It is noteworthy that anti-cancer investigations of CDs reported generally involved two approaches, i.e., *in vitro* and *in vivo* studies. *In vitro* studies are used as the minimal means of assessing the cytotoxicity of CDs. All studies reported here have proven the ability of CDs to induce cellular death. They exert such activity because they can enter the cell via interaction with receptors in cancer cells. However, such effective interactions are possible only if the CDs have surface functional groups that could bring about the desired activity.

In addition, *in vivo* investigations using mice have shown the ability of CDs to reduce tumor weight. Interestingly, the CDs showed significantly lower side effects 14,107. The CDs were assumed to enter the cancer cell passively due to enhanced permeability and retention (EPR) influence 14. However, a recent study that employs cancer membrane (CM) to encapsulate drug-loaded CDs for targeting cancer cells has explicitly shown the influence of surface moieties 107. Thus, although CDs can enter cancer cells passively, the role of functional groups is more pronounced.

Ultimately, functional groups on the surface of CDs are crucial for both *in vitro* and *in vivo* anti-cancer activity. Also, selectivity could be enhanced by attaching targeting substances like monoclonal antibodies and aptamers 108. As such, the surface functional groups could also offer sophisticated treatment strategies for tumors, especially during clinical trials. Finally, *in vitro* and *in vivo* anti-cancer studies obtained from the literature will be summarized in the section.

## Cytotoxic Carbon Dots from Pure Organic Compounds

Synthesis of CDs from specific organic compounds showed immense benefits for targeting cancer cells. For instance, transferrin derived CDs helped target transferrin receptors for cancer drug delivery 15. Thus, carbon dots from molecules capable of inducing cancer mortality could be helpful for enhanced cancer therapy. Thus, reports of CDs with anti-cancer activity are summarized in this subsection **(Table 1).**

Firstly, citric acid and ribonuclease A were used to produce CDs with cytotoxicity against human gastric cancer cell lines (MGC-808). However, information about the specificity of the RNase-A**@**CDs was not made clear despite its relevance on the potential for its application in cancer therapy 13. Furthermore, ginsenoside re is a natural product with antineoplastic properties 109. Carbon dots derived from ginsenoside re showed notable cytotoxicity towards various cancer cell lines, including the human melanoma cell line (A375), hepatocellular carcinoma cell lines (HepG2), and breast cancer cell lines (MCF-7). Interestingly, the Re-CDs showed less than 10% cytotoxicity towards normal cell lines, including human embryonic kidney cell lines (293T), breast cancer cell lines (MCF-10A), and normal liver cell lines (HL-7702) 110. In addition, methotrexate is an anti-cancer drug widely used in chemotherapy. Due to the antineoplastic property of methotrexate, it was used to produce carbon dots with the anti-cancer property. As expected, Methotrexate-derived carbon dots (MTX-CDs) showed anti-cancer potential against tested MDA-MB-231 breast cancer cell lines 111. CDs were synthesized from 1,7'-Dimethyl-2'-Propyl-1H,3'H-[2,5']-bibenzoimidazolyl (DPBI), a compound of valuable biological importance. With the aid of X-ray photoelectron spectroscopy (XPS) and Fourier transform infrared spectroscopy (FTIR), the small-sized CDs (between 2-3nm) were shown to possess various functional groups including hydroxyl (O-H) and carboxylic (COOH) groups. Anti-cancer investigation on these DPBI-CDs compared to standard drug paclitaxel and the DPBI molecule has shown intriguing outcomes. The CDs exerted concentration-dependent cytotoxicity towards MDA-MB-231 at concentrations 25, 50, 75, and 100 μg/mL with 85, 91, 98, and 100% cytotoxicity at the respective concentrations resulting in better than standard drug paclitaxel tested at the same concentrations (81, 88, 95 and 100% cytotoxicity). Furthermore, the CDs were selectively cytotoxic to the tested MDA-MB-231 by exhibiting low toxicity towards red blood cells (RBCs) and white blood cells (WBCs) 112. Curcumin was used as CDs source owing to its well-established anti-cancer activity to target cancer cells. The CurCDs possessed an average diameter of around 4 nm and a surface rich in functional groups originating from the starting material. The antiproliferative study showcased the ability of CurCDs to cause glioma (C6) cell death. Anti-cancer activity of the CDs was comparably similar to that of the curcumin tested. However, CurCDs showed better selectivity towards C6 cells, as proven by the significantly lower toxicity toward HDF cells. The CDs were able to induce the death of the glioma cells by apoptosis 79. Therefore, the CDs derived from curcumin provided the additional advantage of enhanced specificity. As mentioned in the second paragraph of the main section, resveratrol was used to develop CDs as anti-cancer agents. The resveratrol CDs were highly cytotoxic towards cancer cell lines (SH-SY5Y; HCT-116; PC3) while behaving non-cytotoxic towards normal cell lines (HEK-293) 105. Lately, Difluoromethylornithine (DFMO) has been used to develop highly cytotoxic carbon dots towards two kinds of human neuroblastoma cancer cell lines (SMS-KCNR and SK-N-AS). The DFMO-derived CDs could inhibit half of SK-N-AS cell lines at a concentration that is more than fifty times lower than the conventional DFMO drug. Similarly, the concentration of DFMO-CDs required to inhibit 50 percent of SMS-KCNR cell lines was over 30 times lower than that of conventional DFMO required to inhibit the same quantity of cell lines and superseded conjugated DFMO on non-toxic black carbon dots (BCD-DFMO) 113. However, the latest research that falls under the focus of this review reports an astonishing observation 114. Here, CDs were synthesized by two synthetic approaches **(Figure 2)**: hydrothermal and microwave. Based on their synthesis method, these CDs produced from glucose showed overwhelmingly specific anti-cancer activity. CDs prepared by hydrothermal at the temperature of 200ºC and 160ºC for 5 hours showed particular anti-cancer activity against SMS-KCNR while non-toxic towards SK-N-AS and normal cell lines, HEK-293 and human mesenchymal stem cells (MSC). The anti-cancer activity was linked to the synthesis method because similar CDs prepared from microwave synthesis showed no observable toxicity towards both cancer and healthy cell lines 114. Overall, it could be observed that a few things are now more obvious. First, CDs could serve as an anti-cancer agent against various cancer cell lines. Second, the sources of these CDs played a crucial role in determining the potential of the synthesized CDs. Third; the synthesis method could influence the toxicity of the CDs even when a non-anti-cancer agent is used as the precursor. Finally, most often, these CDs' activity supersedes their precursors' activity.

Most of the abovementioned research focuses more on the *in vitro* anti-cancer study. On the contrary, not as many *in vitro* anti-cancer studies reported are available for *in vivo* studies. Nevertheless, the anti-cancer activity of CDs *in vivo* thus far has provided yet insightful information regarding the potential of CDs for tumor therapy. Cancer toxic RNase-A@CDs 13 was shown to be potent for *in vivo* tumor imaging. Interestingly, this research reported the ability of these CDs to be excreted through urine. Therefore, if they were proven to be cytotoxic *in vivo,* as were proven *in vitro,* these CDs would have been more considerable for potential cancer therapy. More intensely, carbon dots obtained from N-hydroxyphthalimide (CD-NHF) resulted in reduced tumor growth (4T1) and metastasis in Balb/c mice.

**Figure 1:**
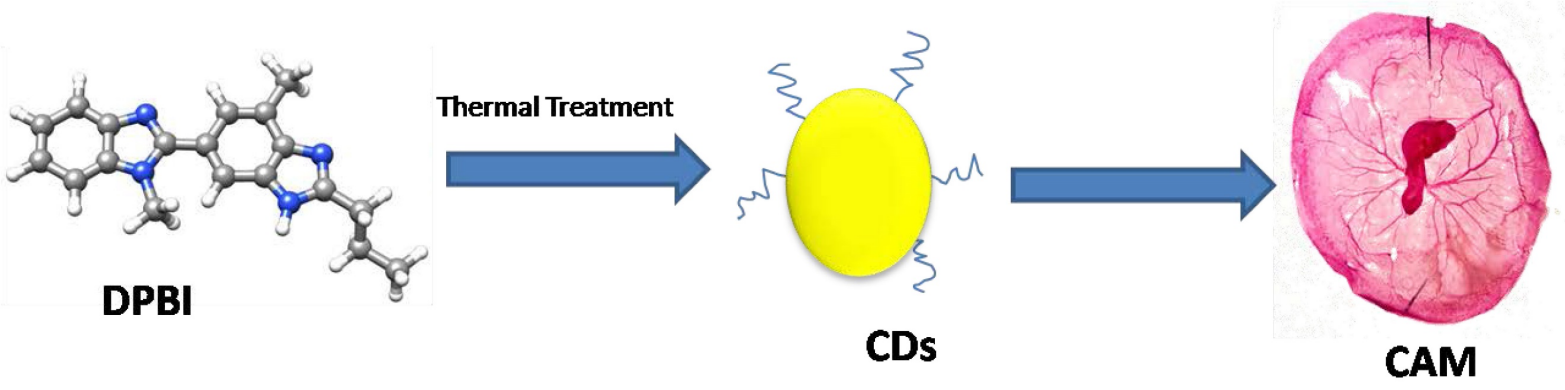
CDs derived from DPBI showed cytotoxicity in an *in vivo* model *ex ovo* chick CAM 112.

**Figure 2:**
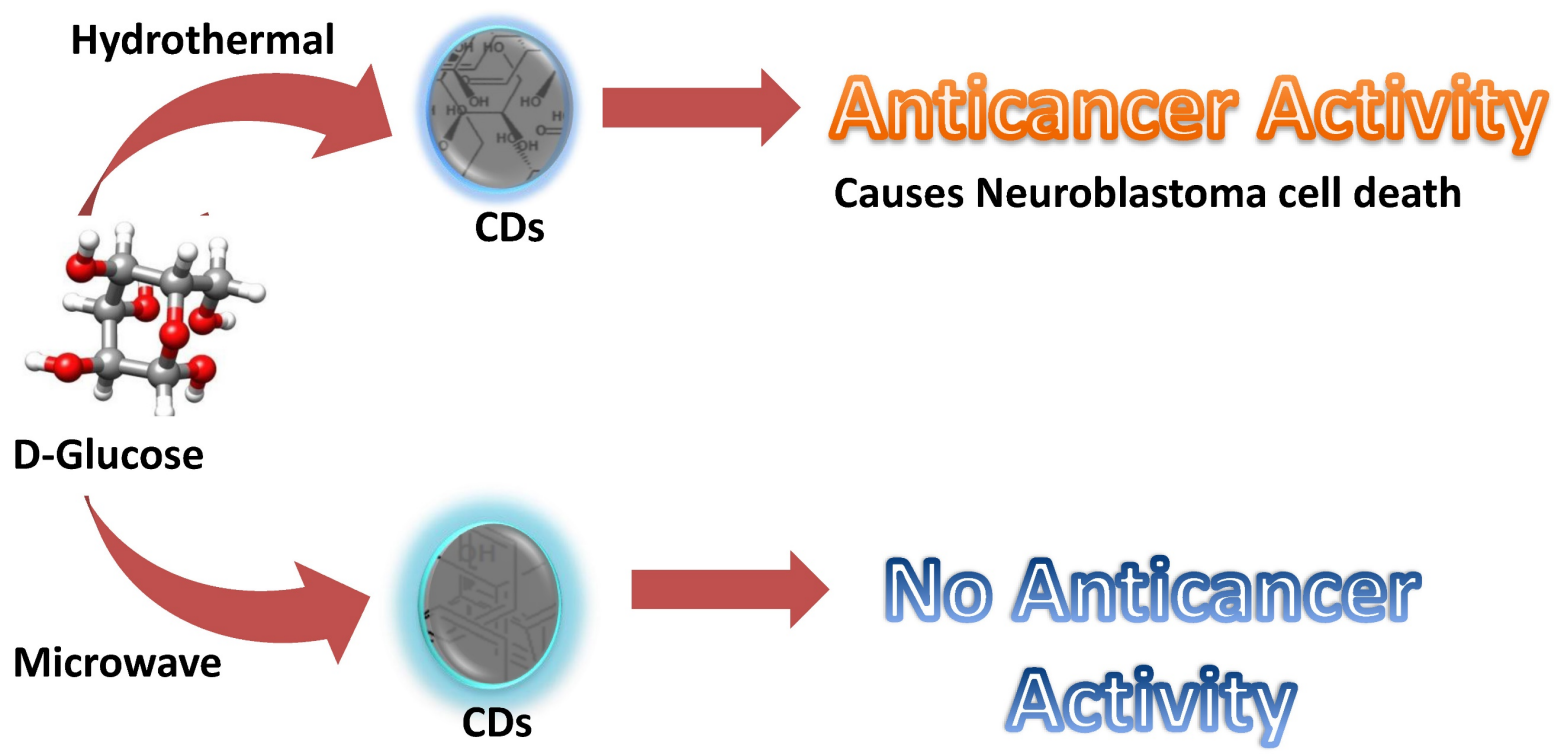
Glucose derived CDs with different application due to difference in synthesis method 114.

The 4T1 cell line is a highly invasive and metastatic breast cancer cell line. Despite its invasiveness, the CD-NHF lowered its growth and inhibited the metastasic nature of these cell lines. Comparatively, untreated groups with 4T1 injected into them experienced additional weight and widespread lung metastasis 85. *In vivo* investigation was conducted by developing a model comprising *ex vivo* chick chorio allantoic membrane (CAM) **(Figure 1)**. Similar to *in vitro* studies, the CDs destroyed the tumor without destroying CAM, as average growth and development were noticed in the treated CAM, whereby limbs and eyes developed 112. So far, *in vivo* studies of CDs having *in vitro* cytotoxicity towards cancer cell lines requires more investigation because it could be possible that CDs might show potency *in vitro* but, in the end, would show poor *in vivo* activity as implied by the abovementioned research 13 that showed the application of CDs for cancer therapy *in vitro.* However, they further limited the application to *in vivo* imaging potential. This phenomenon suggests the inability of the CDs for *in vivo* anti-cancer activity. Overall, *in vivo* studies are crucial to advance CDs for clinical applications. However, up to now, few *in vivo* studies have been reported. It is worth mentioning that a number *in vivo* studies of CDs used in Photothermal therapy have been reported 115-118. As this review focuses more specifically on CDs with innate cytotoxicity towards cancer cells, mentioning those studies has gone beyond the scope of our research.

In summary, the entire CDs possessing anti-cancer activity described above have a common source. They are either obtained from synthetic organic compounds or natural products. Therefore, it could be assumed that forming CDs with anti-cancer activity requires using a source with bioactivity similar to what the CDs are expected to be.

## Cytotoxic Carbon Dots from Organic Crude Sources

Crude organic sources also yielded CDs' cytotoxic activity towards varying cancer cells. A common ground for these sources was the phytochemicals with previously known potential anti-cancer activity. Thus, in this section, we summarized these CDs and their cytotoxic activity **(Table 2).**

The first noted cancer-inhibitive behavior of carbon dots was reported by Hsu and co-researchers 119. In this research, green tea waste-derived carbon dots having an overall negative surface charge showed specific cytotoxicity towards breast cancer cell lines (MCF-7 and MD-MB-231) and human cervical cancer cell lines (HeLa). This condition was suggested by their inability to confer cytotoxicity towards regular breast cell lines (MCF-10A) and standard porcine kidney cell lines (LLC-PK1) 119. In addition, the juice obtained from ginger was used to produce CDs with high specificity toward liver cancer cell lines (HepG2). The CDs surprisingly showed very low cytotoxicity towards other cancer cell lines (A549; MDA-MB-231; HeLa) and normal breast cell lines (MCF-10A), and even normal mouse liver cell lines (FL83B). This form of CDs showed high cytotoxicity towards liver cancer cell lines while having lower cytotoxicity towards other cell lines 14. It suggests the relevance of the structural arrangement of the CDs in their biological activity. Furthermore, *Simarouba glauca* leaves synthesized multipurpose CDs to detect Doxycycline antibiotics. In addition, the *S. glauca* derived CDs were cytotoxic toward breast cancer cell lines (MCF-7). Therefore, the CDs have additional potential in cancer therapy, requiring further investigations 120.

**Figure 3:**
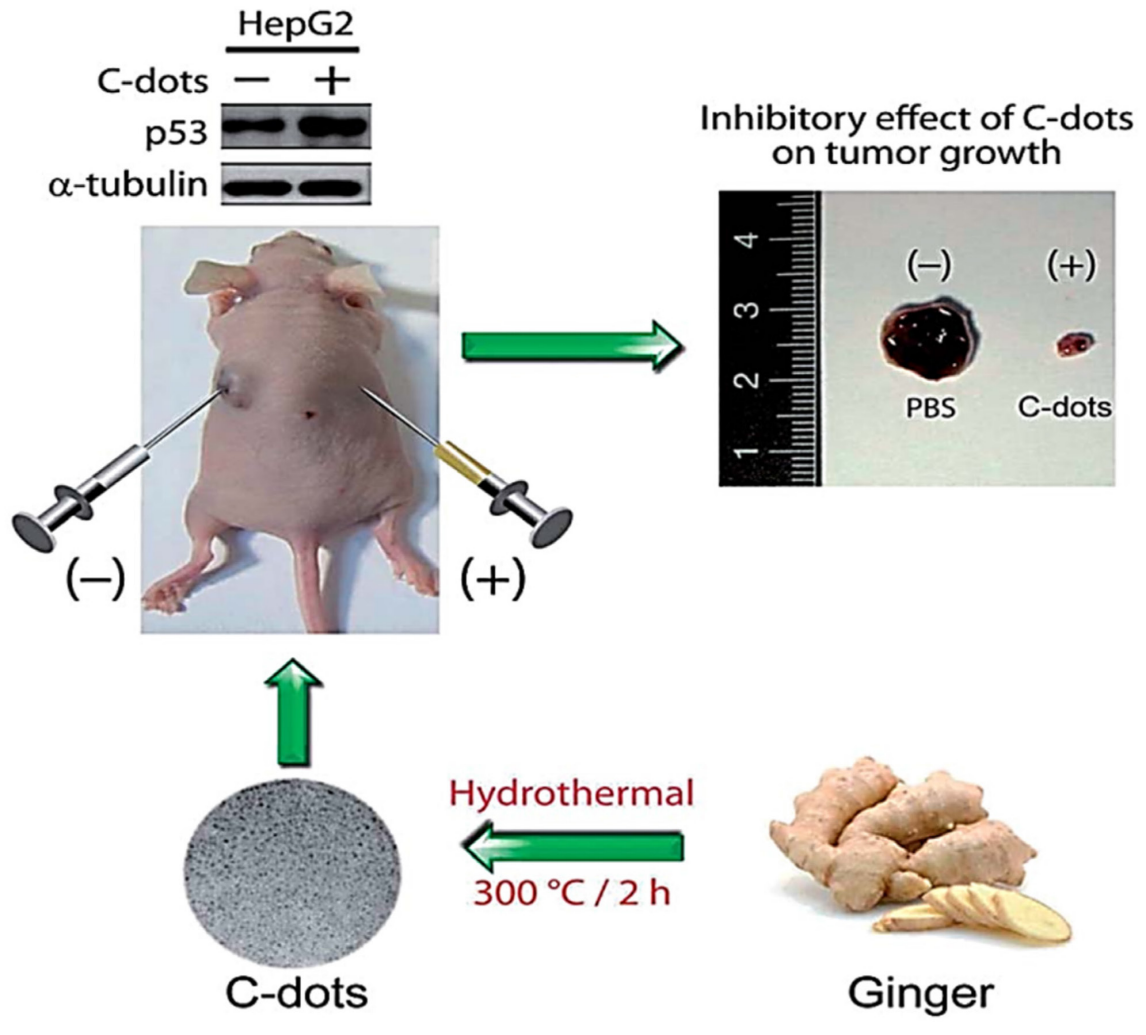
Representation of the fluorescent carbon dots from the ginger extract to inhibit the human hepatocellular carcinoma cell (HepG2). Reproduced from 14 with permission from the Royal Society of Chemistry.

Moreover, CDs obtained from the leaf extract of *Nerium oleander* were exploited for potential anti-cancer activity against MCF-7 cell lines. The CDs had an average diameter of 3.6±0.7 nm and a surface covered with predominantly oxygen-rich functional groups. Anti-cancer activity of these CDs with an overall negative charge surface was studied. Compared to *N. oleander* leaf extracts, the CDs exhibited specific toxicity towards MCF-7 while exerting no cytotoxicity on HDF cells 121. Thus, here also, the CDs showed enhanced selectivity while exerting anti-tumor activity, whereas the starting material is cytotoxic to both normal and healthy cells. Furthermore, carbon dots derived from spices showed specific anti-cancer activity. The spices are black pepper, cinnamon, red chili, and turmeric. All the CDs from the four different kinds of spices showed cytotoxicity towards human glioblastoma cell lines (LN-229) while showing poor cytotoxicity towards normal human kidney cell lines (HK-2) 57. Although not many successful *in vivo* studies of these cytotoxic CDs were reported, carbon dots derived from ginger that was previously 14 shown to be cytotoxic towards HepG2 cancer cell lines *in vitro* were further shown to be effective in inhibiting the cell lines significantly *in vivo* by employing nude mice **(Figure 3).** Finally, CDs from crude organic extracts could also possess desirable anti-cancer potency even though the source consists of various organic compounds. Thus, suggesting the sustainable use of organic wastes in developing functional carbon dots.

## Selective Interaction of Carbon dots with Cancer Cells

As immense interests develop in CDs with anti-cancer potency, understanding the pathway(s) through which CDs exert such potency is crucial in order to able to develop more efficient nanotherapeutic materials. Limited data is available that investigates how CDs interact with cellular systems. Predicting the specific means through which CDs exert effective interaction with cancer cells is challenging. Despite the given situation, we studied different data that report the implication of CDs on cancer cells when exposed. Since the varying biological property of CDs was prominent to the surface functional groups they contain 14,119, we assumed that these CDs also impart cytotoxicity to cancer cells similarly to conventional organic molecules. Thus, they could enhance/inhibit some proteins whose presence in significant amounts or absence could be tragic to these cells. CDs interactions with cancer cells resulted in the programmed death of those cells. However, cell death is accompanied by a series of events. Broadly, these events take place in two pathways. They are extrinsic and intrinsic pathways. The intrinsic route arises when factors result from exposure to chemicals, drugs, or oxidative stress. In comparison, the extrinsic pathway arises from external factors like death activator proteins which bind to tumor necrosis factor receptor (TNF-R) proteins 122. Discussing these events' details has exceeded the scope of this write-up.

Nevertheless, some of the observed pathways followed in apoptosis induced by CDs could give an insight into the interaction pattern of these CDs with the cells. Furthermore, comparing the mode of interaction of traditional drugs used in chemotherapy would add to our aim of understanding how CDs interact. It is solely because the surface of CDs contains some organic compounds or surface functional groups capable of binding with the different receptors 14,119.

Majorly observed pathways found to contribute to cell death of cancers were identified to involve the generation of reactive oxygen species (ROS). A surplus amount of ROS leads to oxidative stress. As Halliwell 123 defined oxidative stress 124, “it is a serious imbalance between the generation of ROS and antioxidant defenses in favor of ROS, causing excessive damage”. Apoptosis induced by the high ROS level could occur in three pathways 124. These pathways are the mitochondrial pathway, Death receptor pathway, and Endoplasmic reticulum (ER) pathway. Generally, most of the encountered literatures describing the apoptotic mechanisms induced by CDs toxicity followed a similar pathway, although with minor variations. The mitochondrial pathway is the pathway through which these CDs utilize to induce apoptosis. Carbon dots derived from green tea induced the generation of H_2_O_2_ and ROS in cancer cells which were able to trigger apoptosis. It was firmly believed that the apoptosis was induced through caspase-3 mediated pathway 119. Although the authors attributed the induced apoptosis to caspase-3 mediation, it is unclear whether the CDs carry out this process through the mitochondrial or ER apoptosis pathway.

Similarly, ginger-derived CDs induced intracellular apoptosis in cancer cells by elevating ROS and H_2_O_2_. This resulted in the up-regulation of the p53 protein. The authors attributed the apoptosis inducement to one of the secondary metabolites in ginger, curcumin. Curcumin is capable of triggering pro-apoptotic factors 14. CDs prepared from ginsenoside Re also caused an increase in ROS levels. Higher levels of ROS cause oxidative damage and apoptosis. Although the expression of caspase-3 was noticed, it could be possible that other cell death inducers play a role since the expression of caspase-3 decreased with increasing concentration of CDs 110. NP-CDs also induced the death of tumor cells by apoptosis. An increased level of cleaved caspases was confirmed. In support of the above observation, B-cell lymphoma 2-associated X (Bax) protein was expressed in abundance, promoting caspases. In addition, a decreased expression in Bcl-2 was noted. The Bcl-2 suppresses caspase activity. Therefore, their under-expression and overexpression of Bax supported the increased caspase activity.

Besides the apoptosis observed, there is evidence of another kind of cell death, that is, autophagy. However, apoptosis is more pronounced in the observed cell deaths due to persistence in its death even when autophagy is curbed 98. Sulfur-doped CDs induced cellular apoptosis-like those previously discussed through the generation of ROS. The ROS resulted from the irradiation of these CDs after being localized in the cells. Interestingly, the CDs were identified to be located mainly at mitochondria and lysosomes, and the observed effect was selective towards cells containing these CDs. The authors further confirmed that apoptosis occurs through the mitochondria pathway 125. Thus, it could be assumed that these CDs interact with the cells to trigger a series of cascades leading to their death. In addition, available data suggested that the cell death process occurs via the mitochondrial pathway.

More specifically, Tiron *et al.* 85 realized a downregulation of heat shock protein (HSP90) after treatment with CDs-NHF. Therefore, HSP90 inhibitors were considered to possess anti-cancer activity. Furthermore, cancer-specific aggregation was characteristic of HSP90 inhibitors 126. Therefore, it is evident that the ability of these CDs to confer specific activity against breast cancer cell lines was due to their ability to inhibit HSP90 proteins. Disparately, CDs derived from tea could instigate alternative reading frame (ARF) protein 16. The ARF protein can inhibit yes-associated protein (YAP) in the nucleus, inhibiting prostate cancer cell lines. Thus, in such a situation, CDs enhanced ARF's ability to inhibit YAP protein crucial in maintaining the viability 127 of tumor cells. While the actual occurrence of cell death inducement is still a mystery, the above data provides insight into the interaction and mechanism of cell apoptosis due to CDs exposure. Furthermore, both approaches could be tracked down to the mitochondrial pathway of apoptosis inducement.

**Figure 4:**
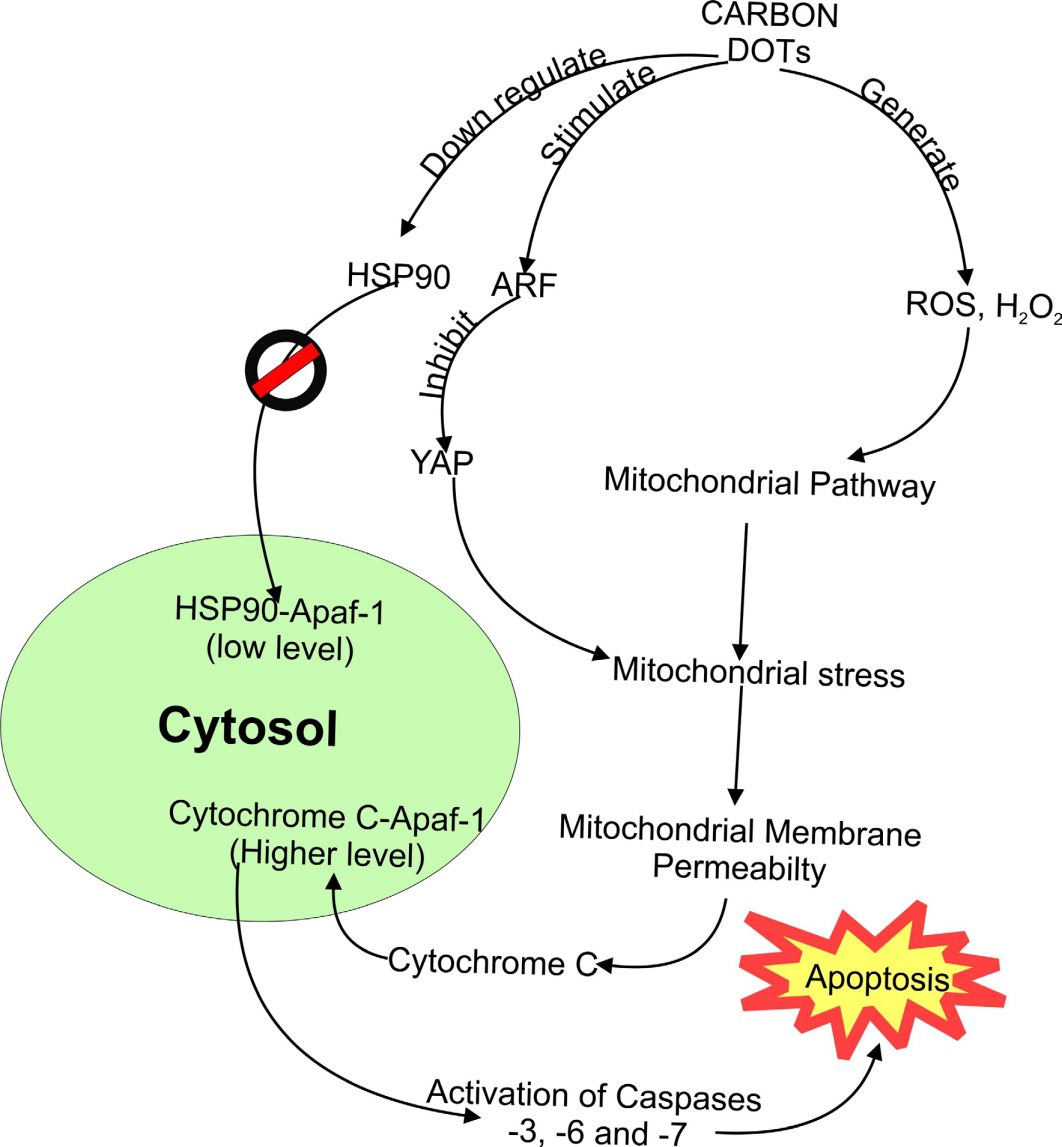
Observed interaction and mechanism of apoptosis in cancer cells due to carbon dots exposure.

In summary as shown in **Figure 4**, CDs as xenobiotic substances enhanced the generation of ROS and H_2_O_2_. However, it leads to mitochondrial stress. Under stressful situations, the permeability of the mitochondrial membrane increases and then releases cytochrome C into the cytosol. In the cytosol, cytochrome C forms a complex with apoptosis activating factor-1 (Apaf-1) and procaspase-9. As a result, mechanical stimulation of caspase-9 was realized. Subsequently, this advances into activating apoptosis executers like caspase-3 124. On the other hand, decreased level of HSP90 was observed after CDs exposure, resulting in the inhibition of cancer cells 85. The HSP90 protein acts by competing with cytochrome c released by mitochondria into the cytosol to form a complex with Apaf-1 (HSP90-Apaf-1). This complex retards the formation of caspases crucial for apoptosis 128. Thus, inhibition of HSP90 by CDs makes the formation of cytochrome c-Apaf-1 complex more efficient with the meager competition.

Similarly, other CDs stimulate alternative reading frame protein (ARF). Interestingly, this inhibits another responsible protein (Yes-associated protein or YAP) 16. The role of YAP is to maintain the mitochondria 127. As such, stimulation of ARF results in lesser YAP, resulting in an apoptosis cascade via the mitochondrial pathway. Although there is still a demand for more data to confirm how these potential cancer-treating CDs cause the selective death of cancer cells, it could be possible that this selective interaction is similar to most xenobiotics in the sense that cells vary in the way they behave after being exposed to foreign toxic substances. Thus, one CD could be fatal to one or more kinds of cancer cells while non-fatal to others. On the other hand, it could be due to the overexpression of different types of receptors in mutated cells (cancer cells), thereby making the carbon dots more specifically bind to these new or over-expressed cellular receptors.

## Limitations in Using Organic Molecules Composing Carbon Dots for Cancer Treatment

As helpful as these CDs seem, developing them for cancer treatment faces several limitations. The starting materials played a significant role in their activity. However, being able to tune or otherwise improve their activity as is done with conventional organic compounds remains difficult. It could be due to the complicated structure these CDs have, making it challenging to study these nanomaterials' structure-activity relationship (SAR). This is mainly due to the inability to orient the surface functional groups of CDs as desired. Since the synthesis involves the aggregation of many similar small molecules into nanosized material, there is still not enough evidence of whether the starting molecules continue to re-orient themselves in the same pattern when the same preparation condition is repeated. It could be more complicated when the crude organic source is used. Thus, understanding the detailed structure of CDs would contribute significantly to finding better cancer treatments.

Consequently, this limits their further application for the therapy. For example, CDs' activity was firmly believed to correlate with their precursor molecules' activity. However, recently, glucose-derived CDs showed different activity, which correlates with the synthesis method instead of starting material. The hydrothermal method yielded CDs with anti-cancer activity, while the microwave-assisted method resulted in non-cytotoxic CDs **(Figure 1)**. It suggests that the two CDs have different structures, although synthesized from the same source. Therefore, CDs also differ based on their structure, indicating possible kinds of CDs similar to organic molecules. Thus, a significant lacking intuition is the knowledge about the exact structural orientation of CDs, which is in turn essential in the CDs' activity.

## Conclusion and Future Perspective

To summarize the review, CDs synthesis could be developed quickly from renewable resources with enhanced activity through either top-down, bottom-up or green synthesis approaches. We have seen that green synthesis offers more sustainable means of CDs development. CDs have a diverse means of preparation ranging from their preparation using low molecular weight organic molecules as well as large molecular weight organic molecules. Interestingly, it was apparent that these CDs' sources influenced their function. Therefore, CDs prepared from a likely anti-cancer organic source would show anti-cancer properties with selective and enhanced anti-cancer activity. In most instances, these CDs interact with cancer cells specifically, resulting in cell death by apoptosis via the mitochondrial pathway. This intuition could provide a solution to the current challenges in cancer treatments.

The future applications of CDs are now apparent. However, the production of these CDs in consideration of their future demands remains unaddressed. Although some carbon dots showed selective cytotoxicity towards some cancer cell lines and less toxicity towards normal cells, extensive study is still needed to estimate the feasibility and druggability of these CDs. Furthermore, the ability to desirably control these CDs' interaction with target proteins is crucial for improving the efficiency of selective cancer targeting. Therefore, understanding the patterns followed by precursors or molecules to carbonize and polymerize into the CDs could open the way for the prediction and engineering of surface functional groups and/or hydrophobic moieties oriented at the “right” position capable of effective interaction with the targeted cellular receptor.

With the number of previously reported literatures 129-137, it is evident that cost-effective, large-scale production of CDs with anti-cancer therapy potential is achievable. Although the large-scale production of CDs with retained therapeutic activity is still awaited, the above literature motivates tuning the reaction conditions in the most optimum way to achieve the large-scale production of these nanomaterials. Furthermore, intense studies on the synthesis methods of CDs could make the development of affordable cancer treatments a reality.

## Figures and Tables

**Scheme 1:**
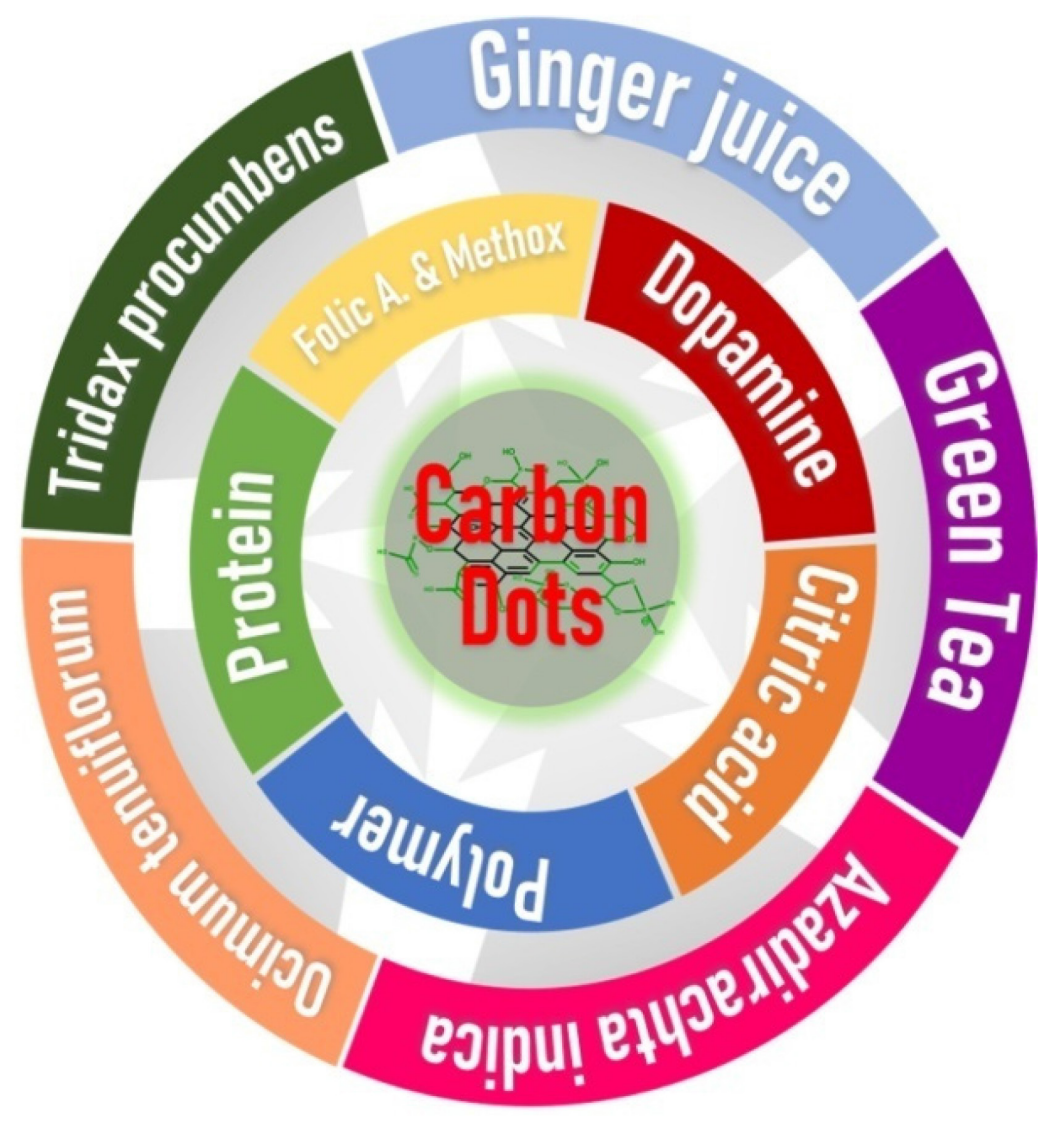
Development of Carbon dots from various organic sources with potential anti-cancer activity.

**Table 1:** Organic compounds used to synthesize cancer specific cytotoxic carbon dots

S/No.	CDs source	Surface Charge (mV)	Cell lines	Cytotoxic?	IC_50_/CC_50_ (**mg/mL)	Ref.
1.	Ginsenoside Re	-	293T (*n)	No	-	110
			MCF-10A (n)	No	-	110
			HL-7702 (n)	No	-	110
			NSFb (n)	No	-	110
			MCF-7	Yes	0.35	110
			A375	Yes	0.22	110
			HepG2	Yes	1.1	110
2.	Methotrexate	+3.97	MDA-MB-231	Yes	3.2μM	111
3.	Resveratrol	-	HEK-293 (n)	No	-	105
			SH-SY5Y	Yes	-	105
			HCT-116	Yes	-	105
			PC3	Yes	-	105
4.	Difluoromethylornithine (DFMO)	+34.7	SMS-KCNR	Yes	0.13μM	113
			SK-N-AS	Yes	0.65μM	113
5.	Citric acid, RNase A	0.02	MGC-808	Yes	-	13
6.	Glucose	-28.7*** -36.4****	HEK-293(n)	No	-	114
			MSC (n)	No	-	114
			SMS-KCNR	Yes	-	114
			SK-A-NS	No	-	114
7.	Curcumin, EDA	-	HDF(n)	No	-	79
			C-6	Yes	-	79
8.	Bibenzonimidazolyl derivative (DPBI)		WBC	No	-	112
			RBC	No		112
			MDA-MB-231	Yes		112

*n= normal cell lines; **Units of IC_50_/CC_50_ reported different from above (mg/mL) are directly mentioned in the table; ***for CDs synthesized at 160ºC for 5 hours by hydrothermal method; ^****^ for CDs synthesized at 200ºC for 5 hours by hydrothermal method.

**Table 2:** Crude organic source used to synthesize cancer specific cytotoxic carbon dots

S/No.	CDs source	Surface Charge (mV)	Cell lines	Cytotoxic?	IC_50_/CC_50_ (**mg/mL)	Ref.
1.	Green tea	-17.2	LLC-PK1 (*n)	No	-	119
			MCF-10A (n)	No	1.75	119
			MCF-7	Yes	0.15	119
			MD-MB-231	Yes	0.072	119
			HeLa	Yes	-	119
2.	Ginger juice	-35	MCF-10A (n)	No	-	14
			FL83B (n)	No	-	14
			A549	No	-	14
3.	*S. glauca*	-	MCF-7	Yes	77.02μL/mL	120
4.	Black Pepper	-24.2	HK-2 (n)	No	-	57
			LN-229	Yes	-	57
5.	Cinnamon	-16.0	HK-2 (n)	No	-	57
			LN-229	Yes	-	57
6.	Red Chilli	-32.9	HK-2 (n)	No	-	57
			LN-229	Yes	-	57
7.	Turmeric	-16.3	HK-2 (n)	No	-	57
			LN-229	Yes	-	57
8.	*N. oleander*	-23.5±6.2	HDF(n)	No	-	121

*n= normal cell lines; **Units of IC_50_/CC_50_ reported different from above (mg/mL) are directly mentioned in the table.
